# Genomic Diversity of Oseltamivir-Resistant Influenza Virus A (H1N1), Luxembourg, 2007–08

**DOI:** 10.3201/eid1509.090452

**Published:** 2009-09

**Authors:** Nancy A. Gerloff, Jacques R. Kremer, Joël Mossong, Matthias Opp, Claude P. Muller

**Affiliations:** National Health Laboratory, Luxembourg, Luxembourg

**Keywords:** Oseltamivir, drug resistance, Luxembourg, H1N1, influenza, viruses, expedited, letter

**To the Editor:** The prevalence of oseltamivir-resistant influenza viruses A (H1N1) (ORVs) increased dramatically worldwide during the winter of 2007–08 (*1*). Recent reports indicated that by early 2009 most influenza virus (H1N1) strains were resistant to oseltamivir (*2*). Resistant viruses were transmitted readily and were as viable and pathogenic as oseltamivir-sensitive viruses (OSVs) (*3*,*4*). The His275Tyr (N1 numbering) mutation in the neuraminidase (NA) genes of influenza virus A (H1N1) that confers resistance to oseltamivir has previously been associated with impaired virus replication, infectivity, and pathogenicity (*5*,*6*).

We investigated the genetic diversity in all 8 gene segments of representative ORVs and OSVs collected during December 2007–March 2008 by the National Influenza Sentinel Surveillance System in Luxembourg (www.lns.public.lu/statistiques/grippe). Phylogenetic analyses were performed by using MEGA version 4.0 (*7*). Tree topology and posterior probabilities were calculated by using MrBayes version 3 (*8*). The sequences have been submitted to GenBank (accession nos. FM174406–60, FN401430–45, and FN401487–FN401518).

Among 140 viruses, 34 strains (24.3%) had the oseltamivir-resistant genotype (Tyr275) in the NA gene. Bayesian analyses of NA genes showed that ORVs formed a distinct cluster supported by high posterior probability (1.00) on the common node (Figure). One resistant strain (LNS-365) was more closely related to OSVs (minimal Kimura distance 0.3%, 4 nt) than to ORVs (minimal Kimura distance 0.5%, 6 nt). In NA protein, 33 ORVs showed the common Asp354Gly substitution in addition to the Tyr275 mutation. The resistant outlier LNS-365 encoded Asp354 like all other OSVs (n = 106). Similarly, only 4 other resistant strains from Europe from the same season shared Asp354 with all 2007–08 sensitive influenza virus (H1N1) strains (n = 251) available in public databases.

**Figure:**
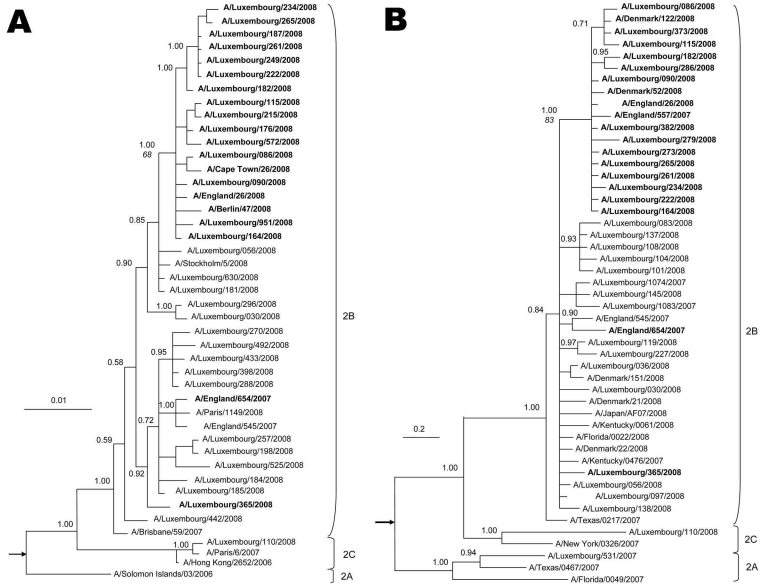
Phylogeny of A) neuraminidase (NA, complete gene) and B) polymerase complex 2 (C-terminal 1,300 nt) genes for selected influenza viruses A (H1N1) from Luxembourg and other countries. Subclades are identified to the right of each tree. The best-approximating model of nucleotide evolution was the general time reversible model with a gamma rate distribution and this model was used for the Bayesian analysis. Markov chain Monte Carlo sampling was implemented in MrBayes version 3 (*8*). In all cases, 6 chains with at least 4 million generations were calculated (10% burn-in removed). At least 2 independent runs of each analysis were performed. Posterior probabilities (indicated on important nodes) of the consensus tree topologies were estimated by sampling likelihood parameters every 125 generations. **Boldface** indicates sequences of oseltamivir-resistant influenza viruses A (H1N1) with the Tyr275 mutation in NA. In MEGA version 4, a neighbor-joining tree with 10,000 replicates was generated to calculate bootstrap values, shown in italics on the node dividing resistant and sensitive strains. Scale bars indicate nucleotide substitutions per site. The trees are rooted on A/New Caledonia/01/1999 and A/BrevigMission/1918 (indicated by arrows).

A total of 18–44 selected sequences from each of the other genes of ORVs and OSVs were generated to investigate which other genetic markers cosegregated with the resistant genotype. Sequences derived from most of the other genes (polymerase proteins PB1 and PA, hemagglutinin, nucleoprotein, matrix protein, nonstructural protein) of ORVs and OSVs were phylogenetically interspersed with no distinct clustering. In contrast, matching the phylogeny of NA, PB2 sequences of genotypically resistant strains (n = 14) formed a distinct cluster supported by high posterior probabilities (1.00) and separate from all OSVs (n = 16) and the resistant outlier LNS-365 (Figure). On the PB2 amino acid level, all OSVs and the resistant outlier LNS-365 shared Pro453, whereas all ORV encoded serine at the same position (Ser453). The outlier LNS-365 differed only by 2 aa from OSVs but by 4 aa from the closest resistant strain.

All published PB2 sequences for influenza virus (H1N1) strains collected since 1918 (n = 720) encoded either Pro453 or His453. Until the emergence of ORVs in 2007, Ser453 was only present in 3 other strains (A/Wilson-Smith/1933 and 2 strains from 1976 and 1988). Located on the surface of the PB2 cap-binding domain (*9*), the Pro453Ser mutation may influence polymerase function and virus replication. The fact that PB2 sequences of ORVs and OSVs are phylogenetically segregated suggests a link between the genetic background and the unexpected fitness of ORVs. There was no amino acid mutation in any of the other genes that segregated in the same way between ORVs and OSVs other than Ser453 (PB2).

Only 1 OSV strain from Luxembourg in 2007–08 (LNS-110) was derived from subclade 2C, unlike the other 139 influenza virus (H1N1) strains (subclade 2B, Figure). Like many other subclade 2C strains, which were recently identified, this virus encoded the amantadine-resistance marker Asn31 in the matrix 2 protein (*10*). Although we did not identify any reassortments between ORVs and OSVs, double-resistant strains may result from co-circulation of amantadine-resistant and ORVs in the same region.

The phylogeny of ORVs identified worldwide (*2*) indicates multiclonal emergence of resistance, which suggests that OSVs may contain low levels of ORV subpopulations. Using pyrosequencing, we determined the incidence and level of mixed alleles in codon 275 of the NA gene (CAT, sensitive and TAT, resistant). In 98 clinical specimens (78 sensitive and 20 resistant strains) no minority alleles were reliably detected above the 3% threshold of the assay. Six OSVs with values between 2.1% and 2.9% were further analyzed by cloning of partial NA genes. No evidence of ORVs was found (Tyr275) in 227 clones.

In summary, we have described amino acid markers in NA (Gly354) and PB2 (Ser453) proteins, which were present in ORVs but absent in all OSVs from Luxembourg in 2007–08. ORVs without this background did not spread as efficiently and were rarely found in Europe. At least 1 resistant virus was more similar to OSV, which suggests >2 clones of resistant viruses in Luxembourg, potentially with different viral fitness. We speculate that the unexpected fitness of the 2007–08 influenza viruses (H1N1) may be caused by a new genetic background that is most likely encoded in the PB2 gene.
